# Humans permanently occupied the Andean highlands by at least 7 ka

**DOI:** 10.1098/rsos.170331

**Published:** 2017-06-28

**Authors:** Randall Haas, Ioana C. Stefanescu, Alexander Garcia-Putnam, Mark S. Aldenderfer, Mark T. Clementz, Melissa S. Murphy, Carlos Viviano Llave, James T. Watson

**Affiliations:** 1 Department of Anthropology, University of California, Davis, Davis, CA, USA; 2 Collasuyo Archaeological Research Institute, Puno, Peru; 3 Department of Geology and Geophysicis, University of Wyoming, Laramie, WY, USA; 4 Department of Anthropology, University of Wyoming, Laramie, WY, USA; 5 School of Social Sciences, Humanities, and Arts, University of California Merced, Merced, CA, USA; 6 Peruvian Register of Professional Archaeologists, Peru; 7 Arizona State Museum and School of Anthropology, The University of Arizona, Tucson, AZ, USA

**Keywords:** archaeology, high elevation, hunter–gatherers, isotopes, bioarchaeology, travel cost analysis

## Abstract

High-elevation environments above 2500 metres above sea level (m.a.s.l.) were among the planet's last frontiers of human colonization. Research on the speed and tempo of this colonization process is active and holds implications for understanding rates of genetic, physiological and cultural adaptation in our species. Permanent occupation of high-elevation environments in the Andes Mountains of South America tentatively began with hunter–gatherers around 9 ka according to current archaeological estimates, though the timing is currently debated. Recent observations on the archaeological site of Soro Mik'aya Patjxa (8.0–6.5 ka), located at 3800 m.a.s.l. in the Andean Altiplano, offer an opportunity to independently test hypotheses for early permanent use of the region. This study observes low oxygen (*δ*^18^O) and high carbon (*δ*^13^C) isotope values in human bone, long travel distances to low-elevation zones, variable age and sex structure in the human population and an absence of non-local lithic materials. These independent lines of evidence converge to support a model of permanent occupation of high elevations and refute logistical and seasonal use models. The results constitute the strongest empirical support to date for permanent human occupation of the Andean highlands by hunter–gatherers before 7 ka.

## 1. Introduction

**Figure 1.**
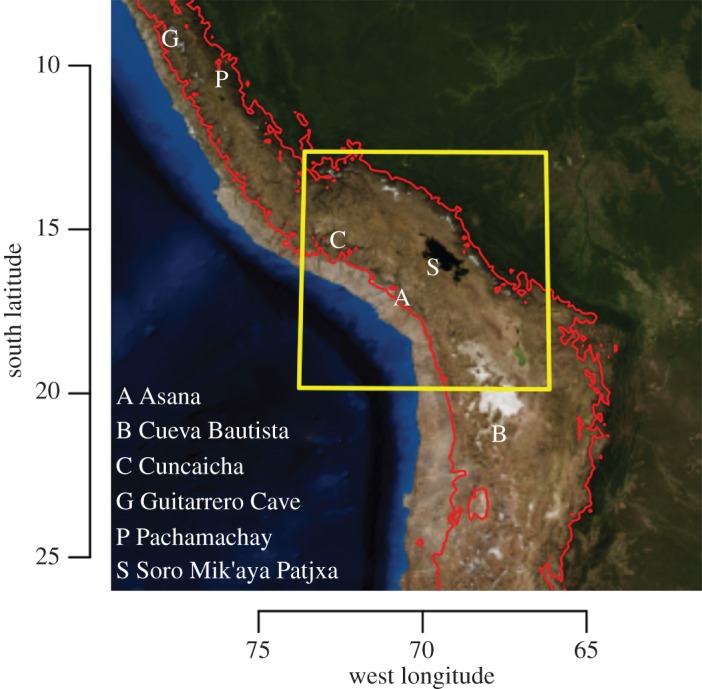
Geographical locations of sites mentioned in text. The red line is the 2500 m.a.s.l. contour derived from a 30 arc-second USGS elevation model [11]. The yellow inset indicates the area of geographical analysis for hypothesis 3. Base imagery from NASA [12].

High-elevation environments above 2500 metres above sea level (m.a.s.l.) pose special challenges for human adaptation [1–3]. Identifying when human populations first began to occupy high elevations in the Andes Mountains on a permanent, super-annual basis is an active area of research and debate [4–6]. Theoretical work has proposed that early Andean hunter–gatherers should have first occupied high-elevation *puna* environments sometime after 9 ka, given the spatial and temporal structure of subsistence resources, especially the post-Pleistocene stabilization of large and predictable vicuña (*Vicugna vicugna*) populations [7–10]. Ethnobotanical and lithic provenance evidence from the site of Pachamachay (figure 1) are consistent with models of permanent highland occupation beginning around 9 ka [8, p. 270]. However, Rick [8] cautioned that ‘The hypothesis of year-round occupation of the Junín puna cannot be conclusively proved with the evidence at hand’. Lithic and settlement pattern evidence from Río Osmore drainage, including the site of Asana, have offered subsequent support for permanent use of the highlands after 9 ka using similar lines of evidence [10]. More recent archaeological findings at the sites of Cuncaicha and Guitarrero Cave may suggest even earlier permanent occupation of the highlands by Terminal Pleistocene hunter–gatherers before 11 ka [4,5,13]. However, Capriles *et al*. [6] contest that the evidence is ambiguous—that seasonal land-use (*sensu* [14,15]) offers an equally plausible explanation for the archaeological observations. Currently then, the best available evidence tentatively suggests that Andean hunter–gatherers first began to permanently occupy high-elevation environments after 9 ka.

Recent discoveries at the archaeological site of Soro Mik'aya Patjxa located at 3800 m.a.s.l. in the Andean Altiplano of Peru [16] offer an independent case study with which to test multiple novel hypotheses related to high-elevation land-use patterns. Excavations produced the remains of 16 human individuals and abundant lithic artefacts from secure pit-feature contexts dating between 8 and 6.5 ka (table 1). Although this context is at least 1000 years later than the posited earliest permanent occupation of the highlands, the findings nonetheless provide a rare opportunity to test multiple previously inaccessible archaeological expectations for early permanent occupation of the highlands by hunter–gatherers.

**Table 1.** Soro Mik'aya Patjxa burials.

provenience					demographic data		isotope chemistry		
burial	feature	area	unit	95% cal. ^14^C date range^a^	sex	age	bone sample	*δ*^18^O_MW_^b^	*δ*^13^C^c^
1	1	1	6	n.d.	ind.	4–6	parietal squama	−13.46	−22.35
2	2	1	7	n.d.	m	30–40	temporal petrous portion	−14.44	−23.95
3	2	1	22	7565–7177	f	18–20	left rib	−16.05	−20.08
4	2	1	22	7565–7177	ind.	12–15	left rib 1	−17.38	−21.24
5	3	1	25	6856–6569	m	50+	right rib	−16.93	−22.49
6	4	7	12	7153–6756	f	35–45	left meta-tarsal 5	−13.81	−21.74
7	10	1	61	6780–6510	f	18–20	left rib	−16.24	−21.14
8	13	1	52	7160–6885	f	30–40	right rib	−14.67	−20.69
9	13	1	48	7465–7317^d^	m	20–30	right rib	−17.50	−21.28
10	14	7	19	6907–6574	f	20–25	thoracic vertebra neural arch	−15.10	−21.76
11	18	7	26	6883–6669	f	25–35	right rib	−15.69	−22.50
12	4	7	9	n.d.	m	35–45	left rib	−12.92	−21.62
13	16	1	55	6829–6565	ind.	4–6	temporal petrous portion	−13.85	−23.73
14	4	7	12	7153–6756	f	30–40	left femur	−14.72	−22.78
15	16	1	58	n.d.	f	35–45	left proximal foot phalanx	−13.86	−23.47
16	18	7	26	7247–7009^d^	m	35–45	left rib 3	−15.66	−22.50

^a^All radiocarbon dates calibrated using SHcal13 [17] using Bchron [18] as implemented in R statistical computing environment [19]. See the electronic supplementary material, table S1 for raw data.

^b^*δ*^18^O_MW (VSMOW)_ reflects inferred drinking water values derived from human bone (see the electronic supplementary material).

^c^*δ*^13^C_(VPDB)_ reflects corrected values for enrichment during carbon incorporation (see the electronic supplementary material).

^d^Dates on human bone. All other dates are on charcoal from associated pit-feature contexts [16].

Soro Mik'aya Patjxa is a hunter–gatherer site that was probably occupied as part of a residentially mobile land-use pattern. Material evidence indicates that the site was not used on a permanent, year-round basis [16]. Within that constraint, we consider three competing models for the site's function and articulation with low-elevation environments. *Model 1* posits that low-elevation populations sent logistical task groups to high elevation to procure high-elevation resources. Soro Mik'aya Patjxa would thus have been a logistical camp accessed from a low-elevation base. *Model 2* posits that transhumant family groups seasonally moved between low- and high-elevation environments as resource availability and weather patterns dictated. Soro Mik'aya Patjxa would thus have been a residential or logistical camp that was part of an annual round including at least a full season above the 2500 m contour as well as below it. *Model 3* posits that forager groups spent the bulk of their annual rounds within high-elevation environments. Soro Mik'aya Patjxa would thus have been a logistical or residential camp that was part of an annual round situated almost entirely at high elevation. Whereas models 1 and 2 envision non-permanent use of the highlands, model 3 envisions permanent use of the highlands. Each of the three basic models leads to a distinct set of archaeological hypotheses. We confront Soro Mik'aya Patjxa with five hypotheses related to human bone chemistry (H1 and H2), geographical positioning (H3), demographic composition (H4) and lithic-material provenance (H5).

These multiple tests can inform ongoing debates about high-elevation land-use patterns—debates that partially revolve around interpretive uncertainties associated with few ambiguous lines of archaeological evidence. Multiple lines of evidence can exponentially reduce—quite literally—uncomfortably high levels of uncertainty. If there is a 20% chance of generating a false positive using one line of evidence and a 20% chance using another, then there is a mere 4% chance (0.20^2^ = 0.04) of generating two false positives with both lines of evidence. The probability of selecting an incorrect model is, therefore, exceedingly small when multiple lines of evidence support the same model. The alignment of evidence afforded by Soro Mik'aya Patjxa would significantly reduce uncertainty in isolating the most likely high-elevation land-use model from the candidate set.

## 2. H1: human bone stable oxygen isotope chemistry (*δ*^18^O)

**Figure 2.**
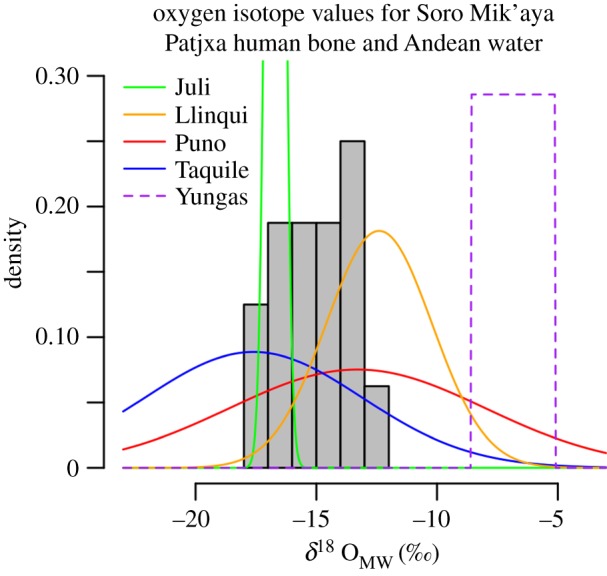
Stable oxygen isotope values (*δ*^18^O_MW_) derived from Soro Mik'aya Patjxa human bone (grey bars) compared with modelled *δ*^18^O_MW_ values for surface water in the Andes (curves) as compiled by Knudson [20]. Solid curves indicate Altiplano water sources approximately 3800 m.a.s.l. The dashed purple line reflects the range of values reported for the mid-elevation Yungas environments, 500–2300 m.a.s.l. The Soro Mik'aya Patjxa samples exhibit values significantly lower than Yungas water but securely within the range of variation for Altiplano surface water. Reported bone values include corrections for well-known fractionation effects (see the electronic supplementary material).

Stable oxygen isotope composition (*δ*^18^O) in the tissues of organisms tends to covary with the elevations of environments in which they live. Knudson's [20] compilation of oxygen isotope values from surface- and groundwater sources in the central Andes shows that surface waters below 2500 m.a.s.l. consistently produce *δ*^18^O_MW (VSMOW)_ (meteoric water) values greater than −8‰ and up to −5‰ (figure 2). Surface- and groundwater sources in the Lake Titicaca Basin around 3800 m.a.s.l. tend to produce lower *δ*^18^O_MW_ values between −25‰ and −8‰. If Soro Mik'aya Patjxa hunter–gatherers permanently occupied elevations above 2500 m.a.s.l. (model 3), we should expect to find *δ*^18^O_MW_ values derived from human bone to fall consistently below −8‰. Logistical use of the highlands from a low-elevation base (model 1) anticipates high values consistently greater than −8‰. Seasonal use (model 2) should produce intermediary values straddling the 8‰ boundary.

These interpretations come with some uncertainty. Past *δ*^18^O saturation levels were not necessarily the same as present levels. However, ice core studies in the southcentral Andean highlands show that Early Holocene *δ*^18^O values were virtually the same as modern values with differences under +2‰ [21]. Diagenetic alternation can also confound interpretation of *δ*^18^O values in archaeological bone carbonates. The examination of carbon and nitrogen mass fractions and carbon : nitrogen (C : N) ratios in the organic fraction of bone can offer some indication of compositional integrity of structural carbonate in the bioapatite because the organic matrix tends to protect bioapatite crystallites [22]. Unaltered bone exhibits bone mass fractions of greater than 35 wt% carbon and 11–16 wt% nitrogen [23–26] with atomic C : N ratios in the range of 2.9–3.6 [27].

### 2.1. Results

Fractionation-corrected stable oxygen isotope values taken from the human bone bioapatite of 16 Soro Mik'aya Patjxa individuals (see the electronic supplementary material) reveal *δ*^18^O_MW_ values between −18 and −12‰ (table 1 and figure 2). These values are consistent with *δ*^18^O_MW_ reported for surface- and groundwater in the Titicaca Basin (approx. 3800 m.a.s.l.) and are inconsistent with values derived from surface waters at elevations below 2500 m.a.s.l. The results therefore support a model of year-round consumption of high-elevation water (model 3) and refute the models of non-permanent use of the highlands (models 1 and 2).

These results are comfortably outside the potential error introduced by palaeoclimatic effects. Bone collagen from burials 9 and 16 produced mass fractions of approximately 44 wt% carbon and approximately 16 wt% nitrogen and C : N ratios of 3.2 and 3.3, respectively. These mass fraction and C : N values indicate little to no diagenetic alteration of the samples.

## 3. H2: human bone stable carbon isotope chemistry (*δ*^13^C)

**Figure 3.**
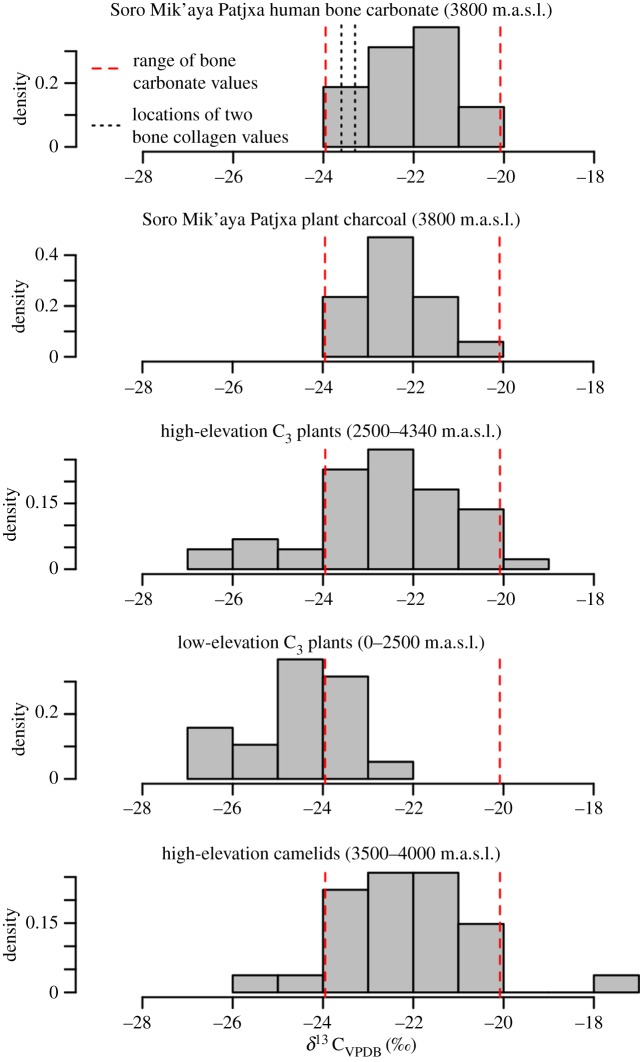
Stable carbon isotope, *δ*^13^C values derived from human bone carbonate from Soro Mik'aya Patjxa (*δ*^13^C_diet_), plant charcoal from Soro Mik'aya Patjxa (*δ*^13^C_plant_), modern C_3_ plants above and below 2500 m.a.s.l. in northern Chile [29], and camelids above 3900 m.a.s.l. in northwest Argentina [32]. Dashed red lines show the range of values for Soro Mik'aya Patjxa human bone carbonate for comparison. All reported values include corrections for well-known fractionation effects (see the electronic supplementary material).

Stable carbon isotope values (*δ*^13^C) in human bone can also offer clues to the structure of mobility patterns across elevational gradients. As elevation increases, *δ*^13^C saturation in the plant tissues that humans directly or indirectly consume increases [28,29]. C_3_ plants sampled between 0 and 2400 m.a.s.l. in northern Chile at 16° S latitude exhibit *δ*^13^C_(VPDB)_ values between −27 and −22‰ [29] including a +1.5‰ correction for post-industrial fossil fuel effects [30,31] (figure 3). By contrast, C_3_ plants between 2500 and 4340 m.a.s.l. tend to exhibit *δ*^13^C_plant_ values between −29 and −19‰ including a +1.5‰ correction for post-industrial fossil fuel effects [30,31]. While there is considerable overlap, low-elevation plants rarely produce values greater than −23‰, and high-elevation plants commonly do. Consistent with the high-elevation values, 17 carbonized plant samples from secure pit-feature contexts at Soro Mik'aya Patjxa produced values between −24 and −20‰ including a +1‰ correction for fractionation owing to carbonization [33]. Modern camelids living above 3500 m.a.s.l. in northwest Argentina and northern Chile exhibit *δ*^13^C values between −28 and −18‰ [29,32] including a +5‰ correction for trophic fractionation [34] and +1.5‰ correction for post-industrial fossil fuel effects [30,31]. The *δ*^13^C values from modern high-elevation plants, archaeological high-elevation plants and modern high-elevation camelids closely align (Kolmogorov-Smirnov test *D > *0.17, *p > *0.42 for all pairwise comparisons), suggesting a consistent high-elevation signature that differs significantly from the *δ*^13^C values of plants in low-elevation ecosystems (*D *= 0.69, *p *<* *0.00).

If the residents of Soro Mik'aya Patjxa used the highlands on a logistical basis (model 1), we would expect *δ*^13^C_diet_ values derived from human bone to fall in the range of −27‰ to −22‰ with values rarely exceeding −23‰. If they used the highlands on a permanent basis (model 3), we would expect to observe values spanning −27‰ to −19‰ with values commonly exceeding −23‰. If they used the highlands on a seasonal basis, we would expect values between −27‰ and −19‰ with a mode around −23‰. As with stable oxygen isotope values, stable carbon isotope values are subject to post-depositional alterations, the extent of which can be assessed by evaluating carbon and nitrogen mass fractions and C : N ratios as described for H1.

### 3.1. Results

Stable carbon isotope values, *δ*^13^C_diet_, derived from the bioapatite of the 16 Soro Mik'aya Patjxa individuals range between −24 and −20‰ (figure 3 and table 1). These values are statistically indistinguishable from the suite of high-elevation samples (*D *= 0.18, *p *= 0.76) and statistically different from the modern low-elevation plant values (*D *= 0.76, *p *<* *0.00). The *δ*^13^C_diet_ values at Soro Mik'aya Patjxa align remarkably well with high-elevation resources and are therefore most consistent with the expectations of permanent use of the highlands (model 3) and are inconsistent with the expectations of non-permanent use of the highlands (models 1 and 2).

All values reported here account for well-known fractionation effects related to carbon incorporation into bone carbonate, carbon incorporation into collagen and carbonization (see the electronic supplementary material). Diagenetic processes are unlikely to confound this interpretation, given the carbon and nitrogen mass fraction values and C : N ratios observed in the collagen of burials 9 and 16 (see H1 results).

## 4. H3: travel distance to 2500 m elevation contour

**Figure 4.**
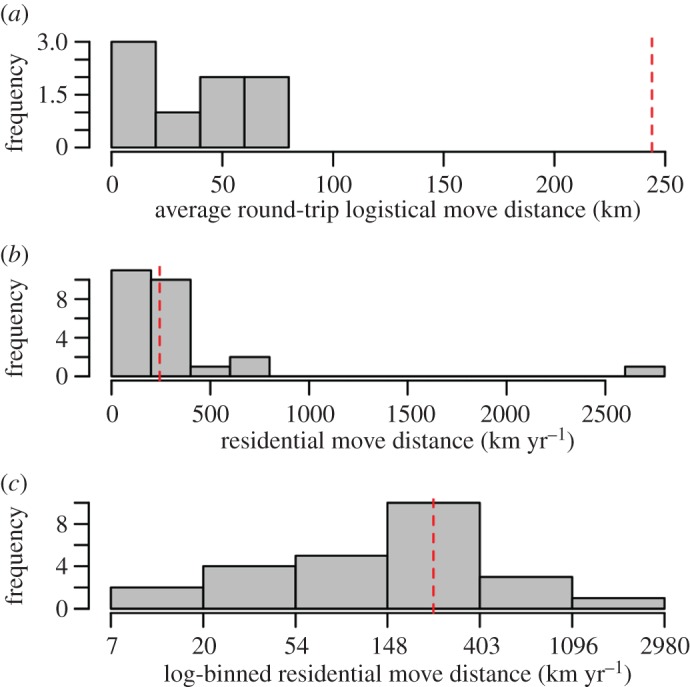
Logistical (*a*) and residential (*b*,*c*) move distances among ethnographic foragers. Logistical moves tend to be less than 80** **km. Total annual move distances average 148** **km and rarely exceed 1000** **km. Adapted from Kelly [35]. Dashed red lines show the minimum round-trip distance between Soro Mik'aya Patjxa and 2500 m.a.s.l. contour.

Kelly's [35] compilation of average logistical move distances among eight ethnographic foraging groups averages 42 km round-trip with all falling under 80 km round-trip (figure 4). Residential move distances for ethnographic foraging groups average 148 km per year with all but one observation falling below 1000 km. Given these ethnographic hunter–gatherer travel distances, if the round-trip distance between Soro Mik'aya Patjxa and the nearest location on the 2500 m topographic contour is greater than 80 km, then the logistical use model (model 1) would be refuted. If the distance is greater than 1000 km, then the logistical and seasonal use models (models 1 and 2) would be refuted. Conversely, if the distance is less than 80 km, then all three models are supported, and if the distance is less than 1000 km, then only the seasonal and permanent models (models 2 and 3) are supported.

### 4.1. Results

**Figure 5.**
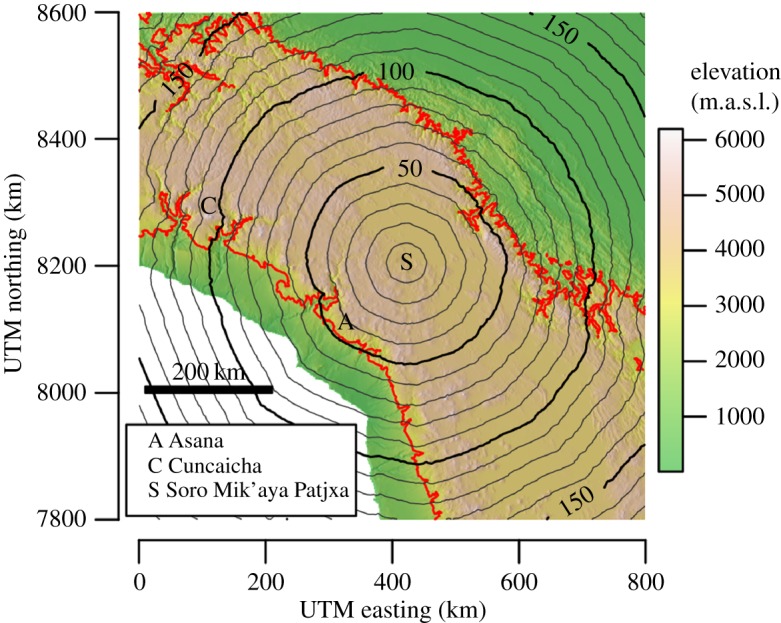
Terrain-adjusted travel times from Soro Mik'aya Patjxa. The isopleths emanating from the site indicate 10 h travel intervals. The red line is the 2500 m.a.s.l. contour. The travel analysis indicates a minimum travel time of approximately 41 h to the nearest location below 2500 m.a.s.l.—a one-way path that traverses 122** **km, or 244** **km round-trip. UTM, Universal Transverse Mercator.

Soro Mik'aya Patjxa is located in the interior highlands of the Andean Altiplano at 16°14′ S, 69°44′ W, 3860 m.a.s.l. (figure 1). Least-cost travel analysis indicates that the minimum one-way travel distance between Soro Mik'aya Patjxa and the nearest location on the 2500 m elevation contour would have been 122 km with a travel time estimate of 41 h (figure 5; see the electronic supplementary material, Material and methods). This translates to a minimum round-trip distance of 244 km—over three times the 80 km maximum logistical move distance observed among ethnographic hunter–gatherers. However, it is within the maximum annual residential move distance. Thus, the geographical evidence suggests that Soro Mik'aya Patjxa was unlikely to have served as a logistical camp tethered to a low-elevation base (model 1). Rather, the geographical position is more consistent with a site that was part of a settlement system that either operated seasonally or year-round at high elevation (models 2 or 3).

## 5. H4: demographic composition

If a given high-elevation site served as a logistical camp operating from a low-elevation base (model 1), we would expect the site's demographic profile to be highly skewed such that children, old adults and either males or females [36]—depending on the logistical task—are under-represented. Specifically, young children between about 4 and 8 years old would probably have been absent from logistical forays, given that they would have been a burden to carry and would have been unlikely to maintain adult walking speeds over long distances even though they might be able to attain such speeds [37]. The ratios of women : men or vice versa would be statistically greater than 50%. By contrast, if a given site was part of a settlement system that was seasonally or permanently situated within that environment (model 2 or 3), we would expect the demographic profile to include the full spectrum of age classes and both sexes in statistical parity (i.e. statistically indistinguishable from 50 : 50).

Although the age profiles derived from burial assemblages tend to be biased reflections of living population structures [38–40], the mere presence of multiple young children or old adults would constitute sufficient evidence for their presence at the site and thus grounds to refute the logistical mobility model (model 1). Sex ratios as a test of the working hypotheses are more sensitive to interpretive error, given that task group organization and burial practices may or may not be gendered. This component of the demographic evidence is therefore interpreted with greater caution.

### 5.1. Results

**Figure 6.**
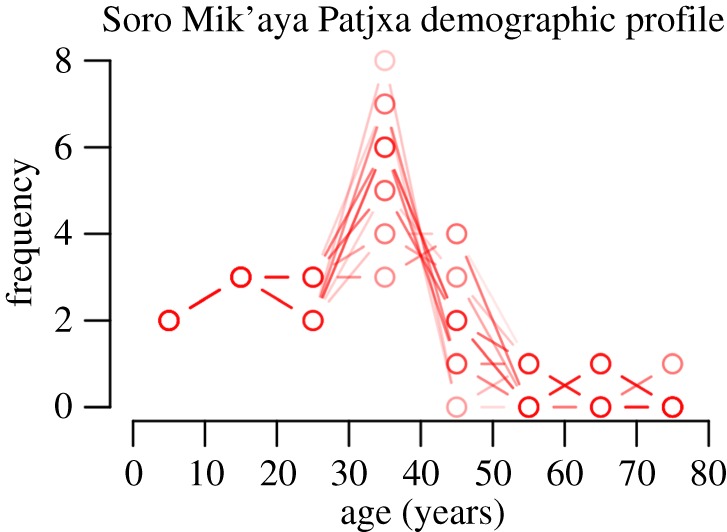
Plausible age-at-death profiles for Soro Mik'aya Patjxa burials given age-at-death estimates for 16 individuals. Histogram binned in 10-year intervals. All age-cohorts are present, suggesting that the Soro Mik'aya Patjxa population does not reflect individuals of logistical foraging groups.

Demographic observations on 16 individuals reveal a profile that is similar to that observed in numerous non-industrial societies [41], including age classes ranging from young children (4–6 years old) to old adults (50+ years old) and a female : male ratio in statistical parity ($x_{1}^{2}=0.69$, *p *= 0.41; figure 6 and table 1; electronic supplementary material). This diverse demographic profile is most consistent with a population that occupied the highlands on either a seasonal or permanent basis (model 2 or 3) and inconsistent with logistical use of the highlands (model 1).

## 6. H5: lithic raw-material provenance

If hunter–gatherers moved logistically into high-elevation environments (model 1), we would expect them to have habitually transported and deposited non-local materials at high-elevation sites such as Soro Mik'aya Patjxa. If they moved seasonally into high-elevation environments (model 2), we would expect low levels of non-local lithic materials. If foragers occupied high-elevation environments permanently (model 3), then we would expect a near absence of non-local raw materials from low-elevation environments [8,10].

The trade of non-local materials could complicate such interpretations, but an absence of non-local materials would nonetheless support the model of permanence. Another complicating factor is raw-material isomorphism across broad geographical extents. While it may be parsimonious to interpret archaeological materials with local analogues as locally acquired, it may be difficult or impossible to completely rule out the possibility of non-local acquisition.

### 6.1. Results

Of the 539 Middle and Late Archaic lithic artefacts examined from the Ilave Basin, 534 (99.1%) were made from raw materials that we located in a raw material survey of the region (electronic supplementary material, table S3). The few samples that did not have clear analogues in our comparative sample included unexceptional materials that may simply reflect extremes in the range of variation in the local materials. The apparent absence of non-local materials at Soro Mik'aya Patjxa and in the Ilave Basin Middle and Late Archaic periods more generally is consistent with year-round use of the high-elevation environment (model 3) and inconsistent with non-permanent use (models 1 and 2).

## 7. Summary and discussion

From three general models for hunter–gatherer use of high-elevation environments in the Andes, we deduced five sets of archaeological hypotheses that we tested against data from the site of Soro Mik'aya Patjxa. All observations are consistent with the expectations for permanent use of high-elevation environments (table 2). No support was found for the logistical use hypotheses, and only partial support was found for the seasonal use hypotheses. We, therefore, conclude that the hunter–gatherers of Soro Mik'aya Patjxa occupied the highlands on a permanent basis by at least 7 ka.

**Table 2.** Summary of three models for hunter–gatherer use of high-elevation environments, five archaeological hypotheses for the Andean highlands and supported results for Soro Mik'aya Patjxa.

	H1: human bone *δ*^18^O_MW_^a^	H2: human bone *δ*^13^C^b^	H3: demographic profile	H4: geographical distance to 2500 m contour	H5: non-local lithic materials
model 1					
logistical use	−8 to −5‰	−29 to −24‰	children and females under-represented	less than 40 km	abundant
model 2					
seasonal use	−12 to −5‰	−28 to −22‰	all ages and both sexes^c^	less than 500 km^c^	present
model 3					
permanent use	−25 to −8‰^c^	−28 to −21‰^c^	all ages and both sexes^c^	any distance^c^	absent^c^

^a^*δ*^18^O_MW_ reflects inferred meteoric water values derived from human bone carbonate (see Material and methods).

^b^*δ*^13^C reflects corrected values for enrichment during carbon incorporation (see Material and methods).

^c^Expectation supported by Soro Mik'aya Patjxa data. Only model 3 finds support across the board.

Archaeological data are notoriously ambiguous, and any one of the tests reported here is subject to varying degrees of uncertainty. Although careful consideration of the data in the light of competing interpretations can go some way towards identifying the most likely explanations [42], alternative explanations may be difficult or impossible to reject entirely. Regarding hypotheses 1 and 2, palaeoclimatic variation and diagenetic processes could potentially explain the observed stable oxygen and carbon isotope values even though the values are more likely to reflect water and food intake by the Soro Mik'aya Patjxa individuals. For hypothesis 3, it is possible that Archaic Period hunter–gatherers made logistical forays to Soro Mik'aya Patjxa from low-elevation base camps even though the extreme distances entailed suggest that long-term use of the highlands was more likely. For hypothesis 4, it is certainly possible that young children trekked over 122 km with more than 1300 m in elevation gain from low-elevation base camps to Soro Mik'aya Patjxa even if that possibility is unlikely. As for hypothesis 5, it is possible that some of the lithic materials examined actually came from distant low-elevation regions even though such an interpretation is less parsimonious than one of local acquisition. Careful consideration of a given line of archaeological evidence can lead to the most likely explanation, but it rarely guarantees freedom from error.

Despite uncomfortably high levels of uncertainty for any one line of evidence, the use of multiple independent lines of evidence can exponentially reduce uncertainty in the greater effort to select the best behavioural models from a candidate set. We considered five lines of archaeological evidence from Soro Mik'aya Patjxa, and although it is difficult to ascribe numerical weight to each result, the convergence of all five lines of evidence on one solution exponentially diminishes the possibility of erroneous model selection. This study, therefore, to our knowledge, offers the most secure evidence for early permanent use of Andean highlands to date and adds to a growing body of empirical support for the theoretical expectation of permanent highland use after 9.0 ka [6,8,10].

A number of other high-elevation sites corroborate the interpretation of early permanent use of the highlands albeit with fewer or less conclusive lines of evidence. Low-elevation lithic materials were rare or absent from the highland sites of the Junín Puna and Rio Osmore regions after 9 ka [8,10]. The geographical positioning of many hunter–gatherer sites in interior, high-elevation locations further opposes the logistical use model (model 1) [43–45]. Such sites are well beyond typical logistical foraging ranges but within annual mobility ranges. Given that at least some of those sites date to the Terminal Pleistocene [45,46], the geographical evidence suggests that hunter–gatherer groups may have begun to use the highlands on at least a seasonal basis beginning in the Late Pleistocene. Archaeological evidence for the timing and structure of hunter–gatherer use of the highlands remains limited, but continued research considering multiple lines of evidence will enhance empirical resolution.

For now, the findings of this study offer a critical parameter value towards modelling rates of cultural and genetic change in the Andean highlands—a region known for the domestication of alpaca, potatoes and quinoa; the emergence of state-level political and economic complexity; and rapid genetic evolution in response to high-elevation stressors. The findings presented here show that estimates for the rates of these cultural and genetic changes can be constrained by a *terminus ante quem* date of 7 ka for permanent occupation of the Andean highlands.

## Supplementary Material

Materials and methods

## Data accessibility

All primary data are presented in table 1 and the electronic supplementary material.
