# Supplementary material for: Humans permanently occupied the Andean highlands by at least 7 ka
Source: R Soc Open Sci. 2017 Jun 28;4(6):170331. doi: 10.1098/rsos.170331 (PMC5493927; doi:10.1098/rsos.170331)
Supplement: Materials and methods [file rsos170331supp1.pdf]

Humans Permanently Occupied the Andean Highlands by at Least 7 ka
Electronic Supplementary Material: Material and Methods

This document presents materials and methods related to the site of Soro Mik'aya Patjxa, carbon and oxygen isotope analysis of structural bone carbonate, carbon and nitrogen isotope analysis of bone collagen, carbon isotope analysis of prehistoric plant charcoal, geographic analysis, demographic analysis, and lithic analysis.

Soro Mik'aya Patjxa archaeological materials

Soro Mik'aya Patjxa is an open-air site covering approximately 2800m^2 in the Andean Altiplano, Peru [1]. Nineteen radiocarbon assays along with material evidence indicate non-permanent use of the site by mobile hunter-gatherers for at least 1000 years between 8.0-6.5 ka (cal. 95%). Seventeen previously reported assays were on charred plant remains recovered from secure pit-feature contexts. Two new assays taken on human bone from two individuals are consistent with the previous results with the new determinations ranging between 7.5 and 7.0 ka (cal. 95%; Table S1). The material assemblage, including over 80,000 artifacts recovered from 50m^2 of excavation, consisted primarily of flaked stone artifacts with lesser amounts of bone, groundstone, red ocher, and charred plant remains. Artifact recovery was primarily achieved with 5mm excavation mesh in plow-zone contexts and 1mm mesh in feature contexts. Ceramics were rare in plow-zone contexts and absent from feature contexts. Sixteen human individuals were recovered, primarily from secure burial pits. At least two pits did not contain human burials, one of which contained large pieces of informal groundstone. Lithic tools consisted primarily of bifaces, especially projectile points consistent with Middle and Late Archaic traditions of the region [2]. Faunal remains consisted primarily of large-bodied mammals—most likely vicuña or taruca.

Carbon and oxygen isotope analysis of human structural bone carbonate

Pre-treatment of 16 human bone samples was undertaken following the procedure published in Koch et al. [3] to remove any secondary carbonates prior to $\delta^{18}O$ and $\delta^{13}C$ analysis. Approximately 1.7mg of treated and pulverized bone bioapatite were loaded into glass vials, sealed with septa then flushed with helium for 5 minutes. After helium flushing 0.5 ml of anhydrous phosphoric acid was added. The samples were reacted with the acid for 24 hours at room temperature. For quality control, internal reference materials (carbonates) were processed at the same time and under the same conditions.

For the analysis of carbonates, the samples were run on a Finnigan GasBench coupled to a Finnigan Delta Plus XP isotope ratio mass spectrometer through an open-split interface at the University of Wyoming Stable Isotope Facility. A reference gas injection system allowed referencing of each sample aliquot to a CO_2 reference gas. Carbon and oxygen isotope values of bioapatite ($^{13}C/^{12}C$ and $^{18}O/^{16}O$) are reported as per mil (‰) normalized to the VPDB scale using in-house carbonate reference materials calibrated against NBS18, NBS19 and LSVEC international reference materials. Analytical reproducibility was assessed by repeated analysis of carbonate reference materials ($\delta^{13}C$ 1 sigma = 0.15‰, $\delta^{18}O$ 1 sigma = 0.08‰). Stable isotope values are expressed in delta-notation and derived using the formula:

Table S1: Radiocarbon age determinations by accelerator mass spectrometry (*n*=19), updated from Haas and Viviano[1].

lab ID	material[a]	provenience				radiocarbon age		95% calendar B.P.[b]		$\delta^{13}C$[c]
		area	unit	level	feature	age	error	max	min	
AA102848	wood	1	61	2	10	5891	49	6780	6510	-21.6
AA102854	wood	7	19	1	14	5914	35	6780	6575	-22.3
AA102843	wood	1	55	1	16	5924	48	6830	6565	-23.7
AA102828	wood	1	25	1	3	5940	49	6855	6570	-21.4
AA102851	bark?	7	11	1	9	5957	48	6875	6640	-23.8
AA102859	wood	7	26	1	18	5983	47	6885	6670	-22.5
AA102858	wood	7	23	1	15	5996	51	6900	6665	-22.3
AA102834	parenchyma	1	33	2	6	6002	48	6900	6670	-22.0
AA102855	parenchyma	7	19	1	14	6003	50	6905	6670	-21.7
AA102837	parenchyma	1	48	1	13	6089	49	7140	6750	-23.9
AA102829	parenchyma	1	25	1	3	6103	48	7150	6760	-22.0
AA102835	twig	1	33	2	6	6148	50	7160	6810	-21.1
AA102842	twig	1	52	1	13	6157	49	7160	6865	-22.3
AA107490	left rib of burial 16	7	26	1	18	6259	38	7245	7010	-17.8
AA102827	grass stem	1	22	1	2	6401	50	7420	7175	-20.9
AA102831	grass stem	1	27	1	5	6458	71	7455	7180	-22.1
AA107345	right rib of burial 9	1	48	1	13	6529	40	7465	7320	-18.2
AA102826	parenchyma	1	22	1	2	6631	50	7565	7430	-23.8
AA102838	twig	1	48	1	13	7090	59	7975	7745	-22.4

[a]All charred materials from lower levels of feature contexts
[b]SHCal13 [17,18]
[c]Values include a +1‰ correction for carbonization fractionation [9]

$$\delta = (R_{sample}/R_{standard} - 1) * 1000 \quad .$$

The $\delta^{18}O$ values were then converted into the VSMOW scale using the published conversion equation of Coplen,

$$\delta^{18}O_{VSMOW} = 1.03091 * \delta^{18}O_{VPDB} + 30.91 \quad .$$

To obtain estimates of drinking water from the bone carbonate oxygen isotope values, we used the published conversion equation of Chenery et al. [4],

$$Drinking\,water = 1.590 * \delta^{18}O_{VSMOW\,(carbonate)} - 48.634 \quad .$$

To account for dietary enrichment during carbon incorporation into the bone carbonates we subtracted 10‰ from the bone carbonate ^{13}C values, which is the mean of the published enrichment values for bone carbonates [5,6].

Carbon and nitrogen isotope analysis of human bone collagen

The University of Arizona Accelerator Mass Spectrometry Laboratory in Tucson performed collagen extraction on two bone samples—one from individual 9 and the other from inidividual 16. The samples were gelatinized by hydrolyzing them in pH 3 hydrochloric acid at 70ºC to pull the collagen into solution.

All subsequent analytical steps were completed at the University of Wyoming Stable Isotope Facility. For each sample, approximately 0.8 mg of dried collagen was loaded into tin capsules. Isotopic counts were obtained on a Costech Elemental Analyzer coupled to a Finnigan Delta Plus XP isotope ratio mass spectrometer through an open-split interface. The weighted samples were dropped into a 1020ºC Cr^3O^3 combustion reactor and pushed along by a helium carrier stream enriched with O_2. The product gases (N_2 and CO_2) were separated on a 3 m Poropak-Q packed gas chromatograph column before entering the IRMS via an open-split interface. Pure reference gases (UHP grade N_2 and CO_2) entered the mass spectrometer at specified times to ensure proper mass calibration.

Carbon and nitrogen isotope values of bone collagen ($^{13}C/^{12}C$ and $^{15}N/^{14}N$) are reported as per mil (‰) normalized to the VPDB and AIR scales, respectively, using in-house materials calibrated against USGS40 and USGS41 international reference materials. In order to account for enrichment during carbon and nitrogen incorporation into bone collagen, we subtracted the enrichment value of 5‰ and 3‰, respectively [5,6].

Bone collagen $\delta^{13}C$ values for the two specimens were -18.6‰ and -18.3‰ and, when compared with their respective structural carbonate $\delta^{13}C$ values, yielded $\delta^{13}C$ values ($\delta^{13}C = \delta^{13}C_{carbonate} - \delta^{13}C_{collagen}$) of 6-7‰—differences that are consistent with omnivorous to more plant-based diets. More importantly, converting collagen $\delta^{13}C$ values to diet values using a standard 5‰ discrimination factor produced $\delta^{13}C$ values within the range expected for plant matter from high-elevation sites (see figure 3, main text).

The percent carbonate of all the bone bioapatite samples was less than expected (~5 wt% expected for modern bone) with values ranging from 0.49-2.63 wt% carbonate. However, the differences in diet carbon isotopic values calculated from raw bone collagen (-23.3‰ and -23.6‰) and bone carbonate (-21.3‰ and -22.5‰) of the two samples were 2‰ and 1‰ ($\delta^{13}C = \delta^{13}C_{diet\ carbonate} - \delta^{13}C_{diet\ collagen}$), respectively, and the atomic or molar C:N ratios of the bone collagen were 3.2 and 3.3, which falls in the acceptable range [7]. Moreover, carbon and nitrogen wt% values were 44 and 16, respectively, which are on par with fresh bone [8]. We therefore consider the bioapatite $\delta^{13}C$ and $\delta^{15}N$ values to have undergone minimal or no alteration.

Carbon isotope analysis of prehistoric plant charcoal

The University of Arizona Accelerator Mass Spectrometry Laboratory in Tucson produced radiocarbon dates with $\delta^{13}C$ values for 17 pieces of charred plant material from secure cultural pit-feature contexts at Soro Mik'aya Patjxa. The values are reported in Table S1 and include a +1‰ correction for carbonization following Turney et al. [9].

Geographic analysis

The location of Soro Mik'aya Patjxa was recorded using multiple readings from a single-frequency global positioning system with an accuracy of approximately 6m. The procedure for

the least-cost path analysis follows that described in Meyer et al [10] with minor modification described here. USGS's 30-m resolution Global Multi-resolution Terrain Elevation Data 2010 (GMTED2010) [11] served as the surface elevation model for cost-path analysis. The area of analysis includes $0-800\,km\,easting$ and $7800-8600\,km\,northing$ (UTM 19S, WGS84). For every DEM cell with a value between 2490 and 2500masl, we calculated the the least-cost out-bound and in-bound travel times to Soro Mik'aya Patjxa. For the cell that produced the minimum round-trip travel time, we then calculated the two-dimensional distance from which we derived the path with the minimal round-trip travel time.

Demographic analysis

Demographic estimates for age and sex were recorded following the standards of Buikstra and Ubelaker [12]. Here we present the determinations for each individual examined.

Burial 1 (F.1/U.1.6) consisted of the fragmented skull of a child of indeterminate sex. Age was estimated between approximately 4 and 6 years age-at-death based on dental development [13]. Deciduous teeth present included the right c1, m1, m1, m2, m2, and the left i2. Wear was mild on the second molar, moderate on the incisor and canine, and heavy on the first molar indicating they had been in occlusion for some time. The developing permanent dentition includes all of the maxillary teeth with the exception of the right I2, left M1 and M3. The mandibular teeth present included both pairs of M1 and I1, and the left P1. Estimation of sex is unreliable in young juvenile remains [14].

Burial 2 (F.2/U.1.7) consisted of the fragmented skull of an adult male. Age was estimated between approximately 30 and 40 years age-at-death based on heavy dental wear [15] and partial suture closure [12]. Secondary sex characteristics including a robust nuchal line and mastoid process, and square chin are indicative of a male individual.

Burial 3 (F.2/U.1.22) consisted of a largely complete adult female. Age was estimated between approximately 18 and 20 years age-at-death based on mild dental wear [15] and several open sutures (including the spheno-occipital synchondrosis). Primary sex characteristics of the pelvis including an open sciatic notch and secondary sex characteristics of gracile occipital and mastoid process morphology were indicative of a female individual.

Burial 4 (F.2/U.1.22) consisted of a partially complete adolescent of indeterminate sex. Age was estimated between approximately 12 and 15 years age-at-death based on epiphyseal union and dental development [12,13]. Estimation of sex is unreliable in juvenile remains [14].

Burial 5 (F.3/U.1.25) consisted of a largely complete older adult male. Age was estimated at approximately 50-55 years age-at-death based on extremely heavy dental wear [15] and several closed sutures. Secondary sex characteristics of the cranium including a robust nuchal crest and mastoid process and prominent glabella and mental eminence were indicative of a male individual.

Burial 6 (F.4/U.7.12) consisted of the partial skeleton of an older adult female. Age was estimated at approximately 35-45 years age-at-death based on heavy dental wear [15]. Secondary sex characteristics of the cranium including a small mastoid process and rounded mental eminence were indicative of a female individual.

Burial 7 (F.10/U.1.61) consisted of the complete skeleton of a young adult female. Age was estimated between approximately 18 and 20 years age-at-death based on dental development [13], mild dental wear [15], minimally closed sutures [12], and a stage 1 auricular surface [16].

Primary sex characteristics of the pelvis including an open sciatic notch, ventral arc, and subpubic concavity, and secondary sex characteristics of gracile nuchal line and glabella were indicative of a female individual.

Burial 8 (F.13/U.1.52) consisted of a nearly complete adult female. Age was estimated between approximately 30 and 40 years age-at-death based on heavy dental wear [15] and a stage 5 auricular surface [16]. Primary sex characteristics from the pelvis included an open sciatic notch, and secondary sex characteristics from the cranium included a gracile nuchal crest and glabella, and were all indicative of a female individual.

Burial 9 (F.13/U.1.48) consisted of the complete skeleton of an adult male. Age was estimated between approximately 20 and 30 years age-at-death based on moderate dental wear [15] and some closure of sutures [12]. Primary sex characteristics from the pelvis including a small preauricular sulcus and narrow subpubic concavity, and secondary sex characteristics including robust cranial and long bone morphology were indicative of a male individual.

Burial 10 (F.14/U.7.19) consisted of the partial skeleton of a young adult female. Age was estimated between approximately 20 and 25 years age-at-death based on moderate dental wear [15] and several minimally closed sutures [12]. Secondary sex characteristics from the cranium include a gracile nuchal crest and intermediate mental eminence, were indicative of a female individual.

Burial 11 (F.18/U.7.26) consisted of the partial skeleton of an adult female. Age was estimated between approximately 20 and 25 years age-at-death based on moderate dental wear [15] and several partially closed sutures [12]. Secondary sex characteristics from the cranium include a gracile nuchal crest and glabella were indicative of a female individual.

Burial 12 (F.4/U.7.9) consisted of a partial adult male. Age was estimated between approximately 35 and 45 years age-at-death based on heavy dental wear [15] and significant closure of several sutures [12]. Secondary sex characteristics of robust mastoid process and supraorbital margin were indicative of a male individual.

Burial 13 (F.16/U.1.55) consists of the fragmented skull of a child of indeterminate sex. Age was estimated between approximately 4 and 6 years age-at-death based on heavy dental development [13]. Estimation of sex is unreliable in young juvenile remains [14].

Burial 14 (F.4/U.7.12)consisted of the partial remains of an adult female. Age was estimated between approximately 30 and 40 years age-at-death based on heavy dental wear [15] and partial closure of several sutures [12]. Secondary sex characteristics of gracile nuchal crest, mastoid process, and supraorbital margin were indicative of a female individual.

Burial 15 (F.16/U.1.58) consisted of the partial remains of an adult female. Age was estimated between approximately 35 and 120 years age-at-death based on heavy dental wear [15] and significant closure of several sutures [12]. Secondary sex characteristics of gracile nuchal crest and glabella were indicative of a female individual.

Lithic analysis

Comparative raw materials were collected from 17 geologic localities throughout the Ilave Basin (Table S2). These localities generally included river gravel beds. At each locale, three individuals test knapped stones for 30 minutes, and medium to high quality stones were collected. The geologic materials were described by geologic origin (e.g., volcanic, sedimentary, or metamorphic), color, and quality.

Table S2: Comparison of lithic raw materials from Middle and Late Archaic cultural contexts to geologic availability in the Ilave Basin. Nearly all raw materials (99.1%) were observed in the local geology.

raw material	Ilave Basin Middle-Late Archaic Points	SMP artifacts			geologic locales[a]
		tools	cores	sum	
black chert	8	2	1	3	3
black fine-grained volcanic	387	12	1	13	14
brown chert	2	0	0	0	1
brownish dull indeterminate[b]	1	0	0	0	0
chalcedony	0	0	1	1	4
gray cherts	3	0	0	0	7
gray fine-grained volcanics	1	0	0	0	4
jaspers	15	1	2	3	7
orange-pink dull indeterminate	1	0	0	0	1
pink chert[b]	0	0	1	1	0
purple fine-grained volcanic	5	2	1	3	2
purple-to-orange cherts[b]	2	0	0	0	0
quartzites	38	7	1	8	10
red fine-grained volcanic[b]	0	1	0	1	0
tan cherts	6	2	0	2	8
tan-and-gray chert	12	0	0	0	3
white fine-grained volcanic?	5	0	1	1	2
white cherts	11	0	0	0	8
indeterminate	6	0	0	0	NA
sum	503	27	9	36	NA

[a]number of geologic locations out of 17—primarily gravel beds—where material was observed.
[b]material not observed in local gravels.

The geologic samples were then visually compared to 489 Middle-Late Archaic lithic artifacts from the Ilave Basin. Projectile point types, their temporal associations, and frequencies are presented in Table S3. The archaeological sample includes 503 Middle-Late Archaic (9-5 ka) projectile points recovered from the surface of various Ilave Basin sites and 36 artifacts including tools and cores from secure pit-feature contexts at Soro Mik'aya Patjxa (8.0-6.5 ka).

References Cited

1. Haas R, Viviano Llave C. 2015 Hunter-gatherers on the eve of agriculture: investigations at Soro Mik'aya Patjxa, Lake Titicaca Basin, Peru, 8000–6700 BP. *Antiquity* **89**, 1297–1312. (doi:10.15184/aqy.2015.100)

2. Klink CJ, Aldenderfer MS. 2005 A projectile point chronology for the South-Central Andean highlands. In *Advances in Titicaca Basin Archaeology-1* (eds C Stanish, AB Cohen, MS Aldenderfer), pp. 25–54. Los Angeles: Cotsen Institute of Archaeology at UCLA.

3. Koch PL, Tuross N, Fogel ML. 1997 The effects of sample treatment and diagenesis on the isotopic integrity of carbonate in biogenic hydroxylapatite. *Journal of Archaeological Science* **24**, 417–429. (doi:10.1006/jasc.1996.0126)

4. Chenery CA, Pashley V, Lamb AL, Sloane HJ, Evans JA. 2012 The oxygen isotope relationship between the phosphate and structural carbonate fractions of human bioapatite. *Rapid Commun. Mass Spectrom.* **26**, 309–319. (doi:10.1002/rcm.5331)

5. Ambrose SH, Butler BM, Hanson DB, Hunter-Anderson RL, Krueger HW. 1997 Stable isotopic analysis of human diet in the Marianas Archipelago, Western Pacific. *Am. J. Phys. Anthropol.* **104**, 343–361. (doi:10.1002/(SICI)1096-8644(199711)104:3<343::AID-AJPA5>3.0.CO;2-W)

Table S3: Surface-collected Middle and Late Archaic Period projectile points from the Ilave region examined for raw material types.

period	projectile point type*	count
Middle Archaic (9.0-7.0 ka)	2B	5
	2C	8
	3B	22
	3E	5
Late Archaic (7.0-5.0 ka)	3F	123
	4D	336
	4G	4
total		503

*Klink and Aldenderfer [2] and Cipolla [19] with calibrated date ranges.

6. Lee-Thorp JA, Sealy JC, van der Merwe NJ. 1989 Stable carbon isotope ratio differences between bone collagen and bone apatite, and their relationship to diet. *Journal of Archaeological Science* **16**, 585–599. (doi:10.1016/0305-4403(89)90024-1)

7. DeNiro MJ. 1985 Postmortem preservation and alteration of in vivo bone collagen isotope ratios in relation to palaeodietary reconstruction. *Nature* **317**, 806–809. (doi:10.1038/317806a0)

8. Harbeck M, Grupe G. 2009 Experimental chemical degradation compared to natural diagenetic alteration of collagen: implications for collagen quality indicators for stable isotope analysis. *Archaeol Anthropol Sci* **1**, 43–57. (doi:10.1007/s12520-009-0004-5)

9. Turney CSM, Wheeler D, Chivas AR. 2006 Carbon isotope fractionation in wood during carbonization. *Geochimica et Cosmochimica Acta* **70**, 960–964. (doi:10.1016/j.gca.2005.10.031)

10. Meyer MC, Aldenderfer MS, Wang Z, Hoffmann DL, Dahl JA, Degering D, Haas R, Schlütz F. 2017 Permanent human occupation of the central Tibetan Plateau in the early Holocene. *Science* **355**, 64–67. (doi:10.1126/science.aag0357)

11. Danielson JJ, Gesch DB. 2011 Global Multi-resolution Terrain Elevation Data 2010 (GMTED2010). Reston,VA, p. 26. (https://pubs.usgs.gov/of/2011/1073/)

12. Buikstra JE, Ubelaker DH, editors. 1994 *Standards for data collection from human skeletal remains: Proceedings of a seminar at the Field Museum of Natural History*. Fayetteville: Arkansas Archeological Survey.

13. Ubelaker DH. 1989 *Human skeletal remains : excavation, analysis, interpretation*. Washington : Taraxacum.

14. Bass WM. 1995 *Human osteology : a laboratory and field manual*. 4th edn. Columbia: Missouri Archaelogical Society.

15. Lovejoy CO. 1985 Dental wear in the Libben population: Its functional pattern and role in the determination of adult skeletal age at death. *American Journal of Physical Anthropology* **68**, 47–56.

16. Lovejoy CO, Meindl RS, Pryzbeck TR, Mensforth RP. 1985 Chronological metamorphosis of the auricular surface of the ilium: A new method for the determination of adult skeletal age at death. *American Journal of Physical Anthropology* **68**, 15–28.

17. Parnell A. 2015 *Bchron: radiocarbon dating, age-depth modelling, relative sea level rate estimation, and non-parametric phase modelling*. See https://cran.r-project.org/web/packages/Bchron/.

18. Hogg A *et al.* 2013 SHcal13 southern hemisphere calibration, 0–50,000 years cal bp. *Radiocarbon* **55**, 1889–1903. (doi:10.2458/azu_js_rc.55.16783)

19. Cipolla LM. 2005 Preceramic Period settlement patterns in the Huancané-Putina River Valley, Northern Titicaca Basin, Peru. In *Advances in Titicaca Basin Archaeology-1* (eds C Stanish, AB Cohen, MS Aldenderfer), pp. 55–64. Los Angeles: Cotsen Institute of Archaeology at UCLA.
